# Introduction of a novel service model to improve uptake and adherence with cardiac rehabilitation within Buckinghamshire Healthcare NHS Trust

**DOI:** 10.1186/s12872-017-0606-2

**Published:** 2017-07-11

**Authors:** Fiona McCartan, Nicola Bowers, Jack Turner, Mirren Mandalia, Nayan Kalnad, Anna Bishop-Bailey, Jiayu Fu, Piers Clifford

**Affiliations:** 1 Buckinghamshire Healthcare NHS Trust, Wycombe Hospital, Queen Alexandra Road, High Wycombe, Buckinghamshire, HP11 2TT UK; 2 Janssen Healthcare Innovation, Janssen-Cilag UK, High Wycombe, UK; 3 pH Associates, Marlow, UK; 4 Janssen Research and Development, Beijing, China

**Keywords:** Coronary heart disease, Cardiac rehabilitation, Emergency readmission, Patient engagement

## Abstract

**Background:**

Buckinghamshire Healthcare NHS Trust (BHT) carried out a cardiac rehabilitation (CR) service redesign aimed at optimising patient recruitment and retention and decreasing readmissions.

**Methods:**

A single centre observational study and local service evaluation were carried out to describe the impact of the novel technology-enabled CR model. Data were collected for adult patients referred for CR at BHT, retrospectively for patients referred during the 12-month pre-implementation period (Cohort 1) and prospectively for patients referred during the 12-month post-implementation period (Cohort 2). The observational study included 350 patients in each cohort, seasonally matched; the service evaluation included all eligible patients. No data imputation was performed.

**Results:**

In the observational study, a higher proportion of referred patients entered CR in Cohort 2 (84.3%) than Cohort 1 (76.0%, *P* = 0.006). Fewer patients in Cohort 2 had ≥1 cardiac-related emergency readmission within 6 months of discharge (4.3%) than Cohort 1 (8.9%, *P* = 0.015); readmissions within 30 days and 12 months were not significantly different. Median time to CR entry from discharge was significantly shorter in Cohort 2 (35.0 days) than Cohort 1 (46.0 days, *P* < 0.001). The CR completion rate was significantly higher in Cohort 2 (75.6%) than Cohort 1 (47.4%, *P* < 0.001); median CR duration for completing patients was significantly longer in Cohort 2 (80.0 days) than Cohort 1 (49.0 days, *P* < 0.001). Overall, similar results were observed in the service evaluation.

**Conclusions:**

Introduction of the novel technology-enabled CR model was associated with short-term improvements in emergency readmissions and sustained increases in CR entry, duration and completion.

## Background

Coronary heart disease (CHD) remains a major cause of morbidity and mortality worldwide; annual estimates indicate that 7.4 million deaths globally and 1.8 million deaths in Europe are attributable to CHD [1, 2]. In the UK, an estimated 2.3 million people have CHD, with approximately 492,000 hospitalisations (324,000 men; 168,000 women) and 69,000 deaths (41,000 men; 28,000 women) attributable to CHD in 2013/14 [3]. Cardiac rehabilitation (CR) is an important component of medical management for CHD, improving longer-term patient outcomes, including readmissions, cardiovascular mortality and quality of life [4–7]. However, despite clear evidence demonstrating beneficial effects, and recommendations for CR in clinical guidelines [8–11], uptake of CR services globally is poor [12–15]. In the UK, the National Audit of Cardiac Rehabilitation (NACR) estimated an overall uptake of CR of 45% in 2013 and 47% in 2014 [12, 16]. Areas identified as requiring action included: low referral rates, uptake and completion rates; prolonged times from index event/discharge to commencement of core CR; inadequate duration of CR programmes; and inadequate pre- and post-CR assessments [12]. NACR emphasised the importance of service review, re-design and innovation to ensure CR programmes meet national recommendations [10–12].

Buckinghamshire Healthcare NHS Trust (BHT) identified the need for a CR service redesign due to poor CR recruitment and completion rates (only 47% of all enrolled patients completed CR and only 5 patients with chronic heart failure were referred for CR annually), and use of paper-based records impaired data-sharing across the CR team. Janssen Healthcare Innovation, a division of Janssen-Cilag UK, supported the patient-centric CR service redesign at BHT with a programme, Care4Today® Heart Health Solutions. The service redesign included development of a web-enabled secure platform designed for healthcare professionals to facilitate patient management along a standardised care pathway, and a patient portal with educational content to increase patient and carer engagement. The novel CR service, implemented in 2013, was aimed at optimising patient recruitment and retention, decreasing readmissions and increasing patient and staff satisfaction. The key characteristics of the pre- and post-service redesign CR programmes are summarised on Table 1.

**Table 1:** Overview of pre- and post-service design cardiac rehabilitation programme characteristics

Cardiac rehabilitation service characteristics	Pre-implementation of Care4Today™ Heart Health Solutions	Post-implementation of Care4Today™ Heart Health Solutions
Records and record keeping	Mainly paper-based records and minimal record keeping - difficult to share across CR teams	Web-enabled secure technology platform providing a standardised and integrated pathway covering: • Patient identification and referral • Referral management and patient recruitment • Comprehensive assessment and personalised programme delivery • Final assessment • Discharge to long-term management
Interface with NACR	Duplication of data entry required for NACR, hospital systems and team records.	Enables uploads to NACR
Liaison between CR healthcare professionals	Informal, ad hoc and fragmented, with poor links with the heart failure team	Facilitates liaison between Cardiac Specialist Nurses and other healthcare professionals, including consultant cardiologists and General Practitioners
Integration of care with community services	Poor links between secondary care and community services	Provides integration between secondary care and community services
First assessments	Minimal first assessments carried out – done at first exercise session (including NACR Questionnaire 1)	Full initial assessment of eligible patients
Final assessments	No final assessments (other than NACR Questionnaire 2)	Full final assessment of eligible patients
Programme individualisation and goal setting	Minimal programme individualisation and goal setting	Provision of individualised programmes and goals for patients and management of patients throughout their CR programme
Educational sessions	Lacked consistency	Includes a full programme of exercise and educational sessions
Patient engagement initiatives	None	Includes patient/carer website allowing access to personalised care plan, progress, educational materials and an additional means of communication with the CR team. Included a patient focus group with feedback received from 122/350 participants.

*CR* cardiac rehabilitation, *NACR* National Audit of Cardiac Rehabilitation

In order to evaluate the impact of the new CR model on service delivery and patient outcomes, a single centre observational study was carried out in conjunction with a local service evaluation. This was carried out by (i) comparing the 12 month periods pre- and post-implementation of the novel CR service in terms of: emergency cardiac-related readmissions within 30 days, 6 months and 12 months of discharge; CR entry, participation and completion; and (ii) assessing patient and staff satisfaction with the new CR service.

## Methods

The observational study and service evaluation were conducted at BHT following implementation of the novel CR service on the 1st October 2013. The observational study and service evaluation involved retrospective data collection for the 12-month pre-implementation period and prospective data collection for the 12-month post-implementation period. The observational study was established to formally evaluate the impact of the service change by comparing pre-implementation (Cohort 1) and post-implementation (Cohort 2) patients (350 patients in each cohort). The service evaluation was carried out in parallel to enable BHT to assess the impact of the service change on an ongoing basis in the interim, and ultimately to collect information in larger groups of patients beyond the scope of the research study.

### Participants

Patients aged ≥18 years treated as inpatients for a clinical cardiac event (index event) either at Wycombe Hospital or surrounding hospitals and referred to BHT for CR were eligible for inclusion in both the observational study and service evaluation (not mutually exclusive). The index event was categorised according to clinical cardiac event: myocardial infarction (MI; including ST-segment elevation MI [STEMI], non-STEMI [NSTEMI] and unknown MI); percutaneous coronary intervention (PCI; including primary and staged PCI; excluding MI); coronary artery bypass grafting (CABG; excluding MI); angina (excluding PCI and CABG); heart failure (HF; excluding PCI); implantable cardioverter defibrillator (ICD; excluding HF); other (all other cardiac events considered appropriate for CR). Patients with unstable HF and patients whose CR spanned the implementation of the novel CR service model were excluded.

The sample size of 350 patients in each cohort for the observational study was estimated based on published UK readmission rates (35%) and modelling of readmission rates expected from uptake of a ‘gold standard’ CR service (24%) [17], and taking into account the minimum reduction in readmissions for cost neutrality for the novel CR service. For the service evaluation, data for all patients meeting the eligibility criteria were included (Cohort 1: *n* = 559; Cohort 2: *n* = 788).

#### Cohort 1 - pre-implementation of the novel CR service model

Eligible patients with an index event occurring between 21st August 2012 and 20th August 2013 were identified retrospectively from BHT’s local copy of the NACR database. For the observational study, consecutive eligible Cohort 1 patients were selected until the number of patients each month matched the number of patients prospectively referred in the corresponding month for Cohort 2 (see below) to ensure that the sample size of 350 patients in each cohort was achieved and matched for seasonal variation. All eligible patients were included in Cohort 1 of the service evaluation (*n* = 559). Cohort 1 patients entered the pre-implementation CR programme between 2nd October 2012 and 26th September 2013 in the observational study and service evaluation.

#### Cohort 2 - post-implementation of the novel CR service model

Consecutive eligible patients with an index event occurring between 22nd August 2013 and 19th August 2014 were identified from BHT’s local copy of the NACR database. The first 350 eligible patients were included in the observational study whereas all eligible patients were included in the service evaluation (*n* = 788). Cohort 2 patients included in the observational study entered the post-implementation CR programme between 2nd October 2013 and 1st May 2015; service evaluation patients entered CR until 21st October 2015.

#### Cohort 2a: Patient satisfaction with the novel CR service model

For the observational study, a subgroup of Cohort 2 patients entering CR from November 2014 consented to complete questionnaires at CR entry and completion. Questionnaires assessed patient expectations and satisfaction with the novel CR service; not all patients answered each question.

#### Staff

Staff involved in the delivery of the CR programme or patients’ acute care at BHT, before and after implementation of the novel CR service, were approached to complete anonymised satisfaction questionnaires. Five staff completed the pre-implementation questionnaire and 5 completed the post-implementation questionnaire; not all staff answered each question.

### Data Collection

Data related to patient demographics, index event, subsequent cardiac-related emergency readmissions, and CR service entry and participation were collected for a minimum of one year from the date of discharge home following the clinical cardiac index event. For Cohort 1, data were collected retrospectively from patients’ paper-based medical records and extracted from the BHT’s local copy of the NACR database. For Cohort 2, data were extracted prospectively from the BHT’s local copy of the NACR database and from the new CR service technology platform (Care4Today® Heart Health Solutions). Extracted data were imported into a bespoke study database for analysis. Patients agreeing to participate in CR following referral were considered to have entered CR. Patients were considered to have completed CR where completion was documented in the medical records (Cohort 1) or where a final assessment date was recorded in the Heart Health System (Cohort 2).

### Statistical analyses

Data were analysed using descriptive and comparative statistics for both the observational study and service evaluation. Quantitative data are presented as arithmetic mean and standard deviation (SD) or median and interquartile range (IQR). Differences in quantitative variables between groups were compared at the two-sided 5% significance level (*P* = 0.05) using the Wilcoxon rank-sum test or Student’s t-test. Categorical data are presented as frequencies (%). Differences in categorical variables between groups were compared at the two-sided 5% significance level (*P* = 0.05) using the chi-squared test. Where variables were compared between more than two groups, a Bonferroni correction was applied to adjust *P* values for multiple comparisons. Analyses were conducted using only the available results with no imputation of missing values; the denominator is reported where data were missing. Data were analysed using Microsoft® Excel and STATA v14.1 (StataCorp LP).

## Results

For the observational study, patients referred for CR in cohorts 1 and 2 were closely matched for age, sex and time from index event to discharge, as shown in Table 2. A higher proportion of patients in Cohort 2 had HF (5.7%) compared with Cohort 1 (1.7%, adjusted *P* = 0.036; see Table 2). Similarly, in the service evaluation, patients referred for CR in Cohorts 1 and 2 were well matched except for a significantly higher proportion of patients with HF in Cohort 2 (6.5%) compared with Cohort 1 (1.8%, adjusted *P* < 0.001; see Table 3).

**Table 2:** Demographic and clinical characteristics of patients referred for cardiac rehabilitation (observational study)

	Cohort 1			Cohort 2		
	Overall	Entered CR	Declined CR	Overall	Entered CR	Declined CR
	(*n* = 350)	(*n* = 266)	(*n* = 84)	(*n* = 350)	(*n* = 295)	(*n* = 55)
Age at index event^a^ (years)	66.3 (11.5)	66.2 (10.9)	66.9 (13.4)	66.7 (11.4)	65.8 (11.4)^d**^	71.1 (10.4)
Male^b^	259 (74.0%)	196 (73.7%)	63 (75.0%)	261 (74.6%)	223 (75.6%)	38 (69.1%)
Index event^b^						
MI	130 (37.1%)	101 (38.0%)	29 (34.5%)	123 (35.1%)	107 (36.3%)	16 (29.1%)
PCI	116 (33.1%)	78 (29.3%)	38 (45.2%)	107 (30.6%)	81 (27.5%)	26 (47.3%)
CABG	41 (11.7%)	35 (13.2%)	6 (7.1%)	46 (13.1%)	42 (14.2%)	4 (7.3%)
HF	6 (1.7%)	4 (1.5%)	2 (2.4%)	20 (5.7%)^e*^	19 (6.4%)	1 (1.8%)
Angina	3 (0.9%)	3 (1.1%)	0 (0.0%)	7 (2.0%)	4 (1.4%)	3 (5.5%)
ICD	0 (0.0%)	0 (0.0%)	0 (0.0%)	1 (0.3%)	1 (0.3%)	0 (0.0%)
Other	54 (15.4%)	45 (16.9%)	9 (10.7%)	46 (13.1%)	41 (13.9%)	5 (9.1%)
Time from index event to discharge^c^ (days)	2.0 (1.0–6.0)	2.0 (1.0–7.0)	1.0 (0.0–4.0)	2.0 (0.0–6.0), *n* = 349	2.0 (0.0–6.0)	2.0 (0.0–6.0), *n* = 54

*CR* cardiac rehabilitation, *MI* myocardial infarction, *PCI* percutaneous coronary intervention, *CABG* coronary artery bypass grafting, *HF* heart failure, *ICD* implantable cardioverter defibrillator

Data presented as ^a^mean (SD), ^b^n (%), or ^c^median (interquartile range), ^d^comparing Cohort 2 patients entering and not entering CR; ^e^adjusted P comparing Cohort 1 and Cohort 2 patients

^*^ *P* < 0.05; ^**^ *P* < 0.01, ^***^ *P* < 0.001

**Table 3:** Demographic and clinical characteristics of patients referred for cardiac rehabilitation (service evaluation)

	Cohort 1			Cohort 2		
	Overall	Entered CR	Declined CR	Overall	Entered CR	Declined CR
	(*n* = 559)	(*n* = 415)	(*n* = 144)	(*n* = 788)	(*n* = 649)	(*n* = 139)
Age at index event^a^ (years)	66.6 (11.6)	66.3 (10.9)	67.7 (13.4)	66.3 (12.5)	65.5 (12.2)^d***^	69.9 (13.3)
Male^b^	414 (74.1%)	305 (73.5%)	109 (75.7%)	590 (74.9%)	489 (75.3%)	101 (72.7%)
Index event^b^						
MI	206 (36.9%)	153 (36.9%)	53(36.8%)	248 (31.5%)	216 (33.3%)^d**^	32 (23.0%)
PCI	173 (30.9%)	112 (27.0%)	61 (42.4%)	256 (32.5%)	191 (29.4%)	65 (46.8%)
CABG	79 (14.1%)	70 (16.9%)	9 (6.3%)	99 (12.6%)	90 (13.9%)	9 (6.5%)
HF	10 (1.8%)	8 (1.9%)	2 (1.4%)	51 (6.5%)^e***^	42 (6.5%)	9 (6.5%)
Angina	5 (0.9%)	5 (1.2%)	0 (0.0%)	15 (1.9%)	9 (1.4%)	6 (4.3%)
ICD	0 (0.0%)	0 (0.0%)	0 (0.0%)	2 (0.3%)	1 (0.2%)	1 (0.7%)
Other	86 (15.4%)	67 (16.1%)	19 (13.2%)	117 (14.8%)	100 (15.4%)	17 (12.2%)
Time from index event to discharge^c^ (days)	2.0 (1.0–7.0) (*n* = 555)	3.0 (1.0–7.0) (*n* = 411)	1.0 (0.0–4.3)	2.0 (0.0–7.0) (*n* = 787)	2.0 (0.0–7.0)	2.0 (0.0–7.0) (*n* = 138)

*CR* cardiac rehabilitation, *MI* myocardial infarction, *PCI* percutaneous coronary intervention, *CABG* coronary artery bypass grafting, *HF* heart failure, *ICD* implantable cardioverter defibrillator

Data presented as ^a^mean (SD), ^b^n (%) or ^c^median (IQR), ^d^adjusted P comparing Cohort 2 patients entering and not entering CR; ^e^adjusted P comparing Cohort 1 and Cohort 2 patients

^*^ *P* < 0.05; ^**^ *P* < 0.01, ^***^ *P* < 0.001

### CR referral and entry

The mean number of CR referrals per month in the pre-implementation period was 40.6 (SD: 9.7) compared with 65.7 (SD: 23.8) in the post-implementation period (*P* = 0.003; service evaluation cohorts).

In the observational study, a significantly higher proportion of referred patients entered CR in Cohort 2 (84.3% [295/350]) compared with Cohort 1 (76.0% [266/350], *P* = 0.006). Similarly, a higher proportion of patients in Cohort 2 of the service evaluation entered CR (82.4% [649/788]) compared with Cohort 1 (74.2% [415/559], *P* < 0.001). There were no significant differences in the demographic and clinical characteristics of patients entering and declining CR in Cohort 1 of the observational study (Table 2) or service evaluation (Table 3). In Cohort 2, those entering CR were significantly younger than those declining CR in both the observational study (*P* = 0.002; Table 2) and the service evaluation (*P* < 0.001; Table 3). In Cohort 2 of the service evaluation, the proportion of patients with MI was significantly higher in those entering CR compared with those declining CR (adjusted =0.004; see Table 3).

### Impact of the novel CR service model on cardiac-related emergency hospital readmissions

#### 30-day readmissions

**Fig. 1:**
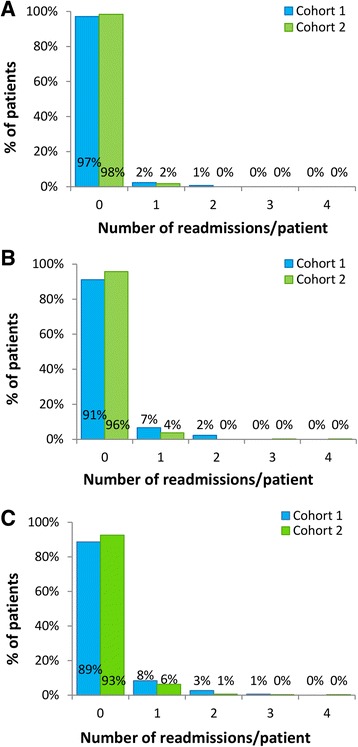
Emergency cardiac readmissions during 12-months of follow-up (observational study). **a** 30-day readmissions; **b** 6-month readmissions; **c** 12-month readmission

**Fig. 2:**
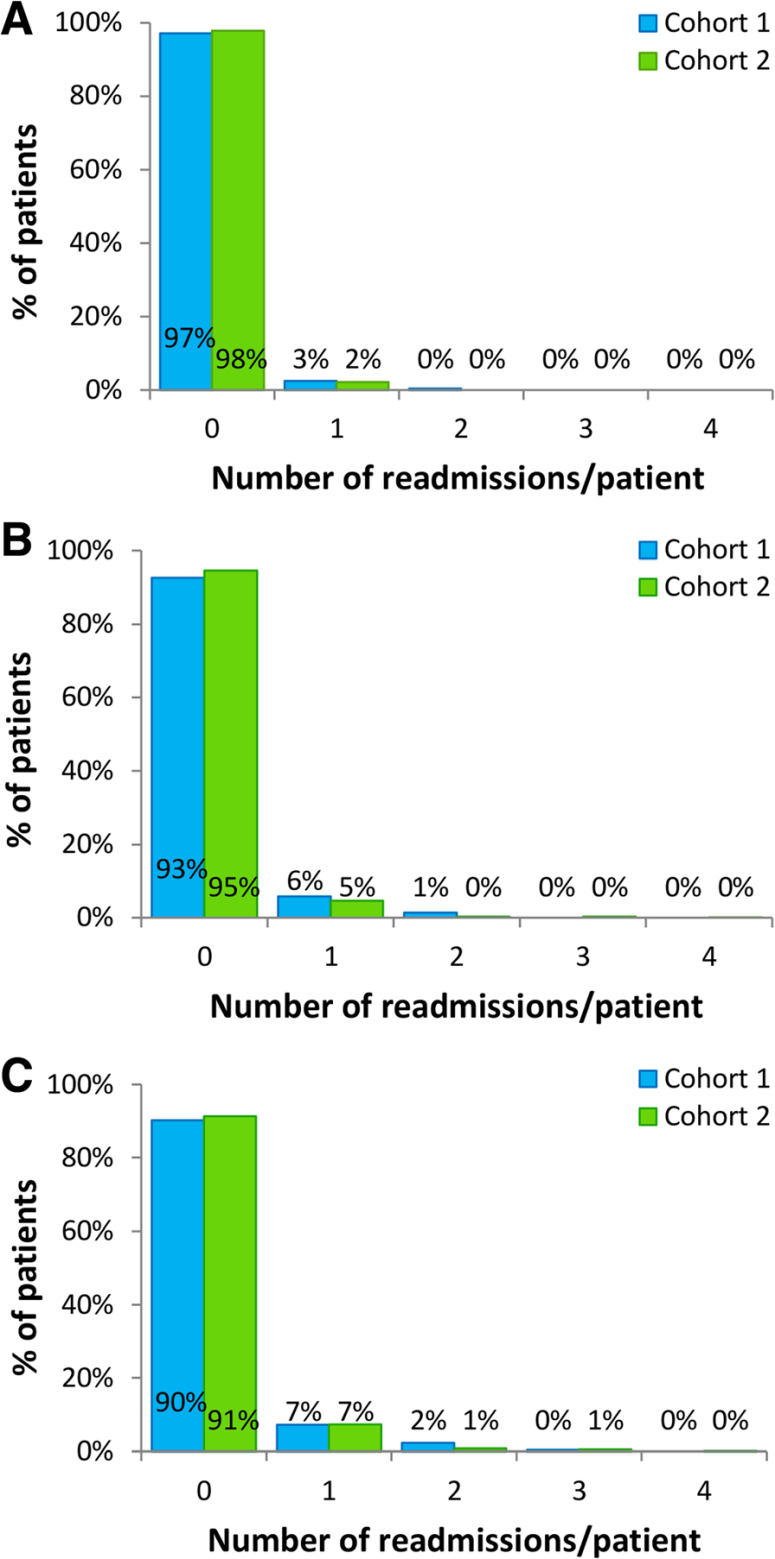
Emergency cardiac readmissions during 12-months of follow-up (service evaluation cohorts)**. a** 30-day readmissions; **b** 6-month readmissions; **c** 12-month readmission

In the observational study, there was no significant difference in the proportions of patients with ≥1 cardiac-related emergency readmissions within 30 days of discharge between the cohorts (Cohort 1: 2.9% [10/350]; Cohort 2: 1.7% [6/350], *P* = 0.31; see Fig. 1a). Similar results were obtained in the service evaluation (Cohort 1: 2.9% [16/559]; Cohort 2: 2.2% [17/788], *P* = 0.41; see Fig. 2a).

#### 6-month readmissions

In the observational study, significantly fewer patients in Cohort 2 had ≥1 cardiac-related emergency readmissions within 6 months of discharge (4.3% [15/350]) compared with Cohort 1 (8.9% [31/350], *P* = 0.015; Fig. 1b). A similar trend was observed in the service evaluation (Cohort 1: 7.3% [41/559]; Cohort 2: 5.3% [42/787], *P =* 0.13; Fig. 2b).

#### 12-month readmissions

In the observational study, there was a trend towards fewer patients with ≥1 cardiac-related emergency readmissions within 12 months of discharge in Cohort 2 (7.4% [26/350]) compared with Cohort 1 (11.4% [40/350], *P* = 0.070; Fig. 1c). The proportions of patients with ≥1 readmissions within 12 months were not significantly different between patients entering and declining CR in Cohort 1 (entered CR: 12.0% [32/266]; declined CR: 9.5% [8/84], adjusted *P* = 1.00) or Cohort 2 (entered CR: 6.4% [19/295]; declined CR: 12.7% [7/55], adjusted *P* = 0.31); there was a trend towards fewer patients with ≥1 readmissions within 12 months in patients entering CR in Cohort 2 compared with patients entering CR in Cohort 1 (adjusted *P* = 0.060). In the service evaluation the proportions of patients with ≥1 cardiac-related emergency readmissions within 12 months were not significantly different (Cohort 1: 55/559 [9.8%]; Cohort 2: 68/784 [8.7%], *P* = 0.47; Fig. 2c) and were not significantly different comparing patients entering and declining CR in Cohort 1 (entered CR: 43/415 [10.4%]; declined CR: 12/144 [8.3%], adjusted *P* = 1.00) or Cohort 2 (entered CR: 52/646 [8.0%]; declined CR: 16/138 [11.6%], adjusted *P* = 0.54) or comparing patients entering CR in Cohorts 1 and 2 (adjusted *P* = 0.59).

#### CR participation

In the observational study, median times to CR entry from index event and from discharge were significantly shorter in Cohort 2 compared with Cohort 1 (see Table 4). The median duration of CR was significantly longer in Cohort 2 (72 days) compared with Cohort 1 (42 days, *P* < 0.001), with similar results in the service evaluation (see Table 5).

**Table 4:** Cardiac rehabilitation participation (observational study)

	Cohort 1			Cohort 2		
	Overall	Completed	Did not complete CR	Overall	Completed CR	Did not complete CR
	(*n* = 266)	CR (*n* = 126)	(*n* = 140)	(*n* = 295)	(*n* = 223)	(*n* = 72)
Time from index to CR entry (days)	51.1 (37.3–70.8)^a***^	52.5 (36.3–71.0)	51.0 (40.0–69.3)^e**^	41.0 (27.0–62.0)	41.0 (27.0–64.0)^d*^	39.5 (23.8–59.0)
Time from discharge to CR entry (days)	46.0 (35.0–63.0)^a***^ (*n* = 253)	47.0 (33.0–65.3) (*n* = 120)	45.0 (37.0–63.0)^e**^ (*n* = 133)	35.0 (23.0–56.0)	36.0 (23.0–56.5)^d*^	34.5 (23.0–51.5)
Duration of CR (days)	42.0 (33.5–56.0)^a***^ (*n* = 263)	49.0 (38.3–69.5) ^b***^	42.0 (21.0–56.0)^e**^ (*n* = 137)	72.0 (50.0–100.0) (*n* = 294)	80.0 (64.0–107.0)^d***^ (*n* = 222)	9.0 (1.0–43.0)^c***^
CR sessions attended	6.0 (4.0–7.0)	6.0 (5.0–8.0)^b***^	5.0 (2.0–6.3)^e***^	6.0 (3.0–8.0)	7.0 (5.0–9.0)^d^	2.0 (1.0–4.0)^c***^

*CR* cardiac rehabilitation

^a^comparing Cohort 1 and Cohort 2; ^b^adjusted P, comparing Cohort 1 patients completing and not completing CR; ^c^adjusted P, comparing Cohort 2 patients completing and not completing CR; ^d^adjusted P, comparing Cohort 1 and Cohort 2 patients completing CR; ^e^adjusted P, comparing Cohort 1 and Cohort 2 patients not completing CR

^*^ *P* < 0.05, ^**^ *P* < 0.01, ^***^ *P* < 0.001

**Table 5:** Cardiac rehabilitation entry and participation (service evaluation). Data presented as median (interquartile range)

	Cohort 1			Cohort 2		
	Overall	Completed CR	Did not complete CR	Overall	Completed CR	Did not complete CR
	(*n* = 415)	(*n* = 183)	(*n* = 232)	(*n* = 649)	(*n* = 481)	(*n* = 168)
Time from index to entry into CR (days)	50.0 (36.0–67.0)^a***^	51.0 (35.0–67.0)	50.0 (37.0–67.3) ^e***^	39.0 (23.0–61.0)	39.0 (24.0–61.0) ^d***^	38.0 (21.8–59.3)
Time from discharge to entry into CR (days)	43.0 (33.0–61.0)^a***^ (*n* = 393)	43.0 (30.0–61.0) (*n* = 176)	43.0 (35.0–59.0) ^e***^ (*n* = 217)	32.0 (21.0–53.0)	33.0 (21.0–55.0) ^d***^	29.0 (20.0–50.3)
Duration of CR (days)	42.0 (28.0–63.0)^a***^ (*n* = 408)	49.0 (35.0–68.5) ^b***^ (*n* = 180)	42.0 (21.0–56.0) ^e***^ (*n* = 228)	71.0 (48.0–97.0) (*n* = 647)	78.0 (64.0–105.0)^d***^ (*n* = 479)	14.5 (1.0–43.0) ^c***^
CR sessions attended	6.0 (3.0–7.0)^a**^	6.0 (5.0–8.0)^b***^	5.0 (2.0–7.0)^e***^	6.0 (2.0–9.0)	8.0 (5.0–9.0)^d***^	2.0 (1.0–4.0) ^c***^

*CR* cardiac rehabilitation

^a^comparing Cohort 1 and Cohort 2; ^b^adjusted P, comparing Cohort 1 patients completing and not completing CR; ^c^adjusted P, comparing Cohort 2 patients completing and not completing CR; ^d^adjusted P, comparing Cohort 1 and Cohort 2 patients completing CR; ^e^adjusted P, comparing Cohort 1 and Cohort 2 patients not completing CR

^*^ *P* < 0.05. ^**^ *P* < 0.01, ^***^ *P* < 0.001

In the observational study, there was no significant difference in the number of CR sessions attended between the cohorts (Table 4), whereas in the service evaluation patients in Cohort 2 attended a significantly higher number of CR sessions than Cohort 1 (*P* = 0.001; Table 5). In the observational study, a significantly higher proportion of patients completed the CR programme in Cohort 2 (223/295 [75.6%]) compared with Cohort 1 (126/266 [47.4%], *P* < 0.001). The duration of CR and number of CR sessions attended were significantly higher in those completing CR compared with those not completing CR in both cohorts (Table 4). The median times from index event and discharge to CR entry were significantly shorter in Cohort 2 compared with Cohort 1 in those completing and those not completing CR (Table 4). For patients who completed CR, the median duration of CR was significantly longer in Cohort 2 (80.0 days) compared with Cohort 1 (49.0 days, adjusted *P* < 0.001; Table 4). There was a trend towards a higher median number of CR sessions attended in Cohort 2 (7.0 sessions) than Cohort 1 (6.0 sessions, adjusted *P* = 0.064; Table 4). For patients who did not complete CR, the median duration of CR and median number of CR sessions attended were significantly shorter in Cohort 2 compared with Cohort 1 (Table 4). Similar results were observed in the service evaluation (see Table 5). Reasons for CR discontinuation were recorded for 37/232 (15.9%) patients in Cohort 1 and 106/168 (63.1%) patients in Cohort 2 of the service evaluation; of the diverse reasons recorded, ‘achieved aims’ and ‘too ill’ were the most frequently recorded (see Table 6).

**Table 6:** Reasons for discontinuing CR (service evaluation)

	Cohort 1 (*n* = 232)	Cohort 2 (*n* = 168)
Achieved aims	14 (6.0%)	7 (4.2%)
Work commitments	3 (1.3%)	4 (2.4%)
Left this area	0 (0.0%)	8 (4.8%)
Hospital re-admission	2 (0.9%)	2 (1.2%)
Planned/emergency intervention	3 (1.3%)	1 (0.6%)
Too ill	10 (4.3%)	14 (8.3%)
Died	1 (0.4%)	4 (2.4%)
Other	4 (1.7%)	66 (39.3%)
Not recorded	195 (84.1%)	62 (36.9%)

Data presented as n (%)

#### Patient and staff satisfaction

**Fig. 3:**
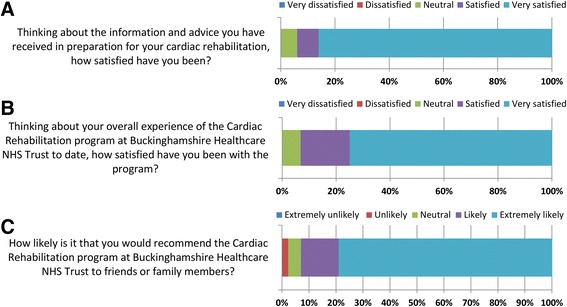
Patient satisfaction with the cardiac rehabilitation service. **a** Patient satisfaction at cardiac rehabilitation entry; **b** patient satisfaction at cardiac rehabilitation exit; **c** family and friends recommendation

Of 50 patients in Cohort 2 completing the CR entry questionnaire, 47 (94.0%) were satisfied/very satisfied with the information and advice received in preparation for CR (see Fig. 3a). Of 44 patients completing the CR completion questionnaire, 41 (93.2%) were satisfied/very satisfied with their overall experience of the CR programme (Fig. 3b). The mean time between completion of entry and exit questionnaires was 111.7 (SD: 34.1) days; 21 (47.7%) patients reported the same level of satisfaction at entry and exit, 9 (20.5%) were more satisfied at exit and 14 (31.8%) were less satisfied at exit compared with entry. The majority of patients (40/43 [93%]) were likely/extremely likely to recommend the CR programme to friends/family (Fig. 3c).

**Fig. 4:**
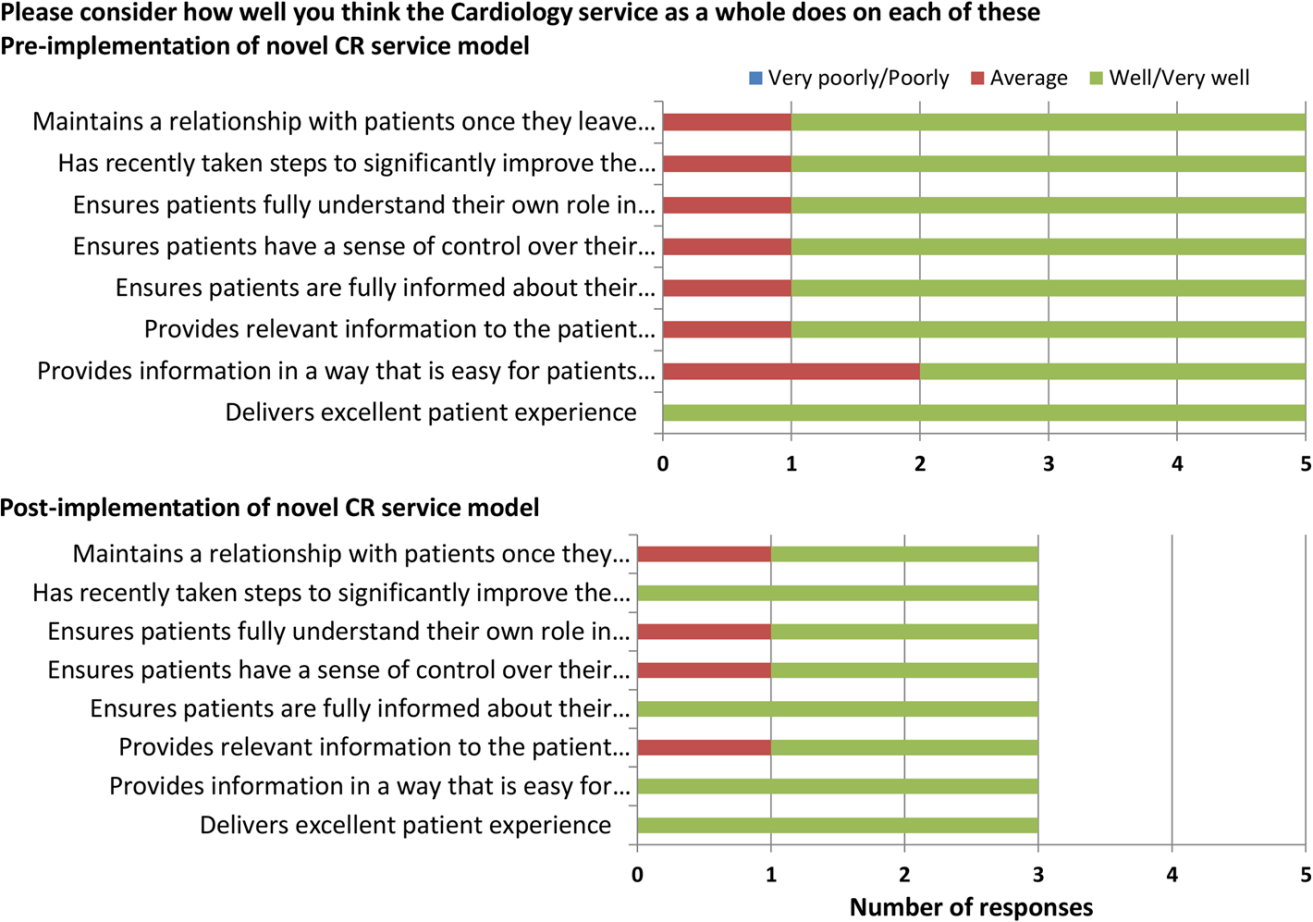
Staff satisfaction with the cardiac rehabilitation service. Staff satisfaction pre-and post-implementation

Of the 5 members of staff completing the question on pre-implementation CR service performance, 80% reported the service did well/very well on all aspects evaluated except provision of information in a way easy for patients to understand (Fig. 4). Of the 3 members of staff completing the question on the post-implementation CR service performance, all respondents indicated that the service did well/very well in terms of: taking steps to improve the quality of patients’ rehabilitation; ensuring patients were fully informed about their rehabilitation; providing information in a way that was easy for patients to understand; and delivering an excellent patient experience (see Fig. 4).

## Discussion

In the UK, CR is considered an important component of integrated cardiology services, with the British Association for Cardiovascular Prevention and Rehabilitation (BACPR) emphasising that referral of eligible patients for CR is as important as prescription of cardioprotective medication [10]. While exercise is a core component of all CR programmes, guidelines recommend more comprehensive programmes, which additionally include a range of educational activities and psychological and social support. However the nature and frequency of different interventions and the overall duration and intensity of CR programmes vary considerably [7, 18]. There is evidence to suggest that differences in the design and implementation of CR programmes, including factors such as supervised exercise, development of individualised programmes, and use of information technology, can influence patient outcomes [19, 20]. Based on the results of the present observational study and service evaluation, introduction of the novel CR service model was associated with short-term improvements in emergency readmissions and increases in: monthly patient referrals to CR, the proportion of patients with HF referred for CR, the proportions of referred patients entering and completing CR, and the duration of CR.

More Cohort 2 patients were referred for CR compared with Cohort 1, suggesting introduction of the new service model increased identification of patients suitable for CR by clinicians. However, we cannot exclude that there was an increase in the number of patients with cardiac events post-implementation of the novel CR model. In both cohorts of the observational study and service evaluation, women represented approximately 25% of all referred patients, with no difference in the proportion of women entering or declining CR. Based on recent UK data, women comprise approximately 34% of all CHD-related hospitalisations [3], suggesting that women were under-represented amongst those referred for CR pre- and post-implementation of the new CR service model. These results are consistent with the results of systematic reviews and meta-analyses of CR clinical trials and recent NACR data indicating under-representation of women in CR programmes is widespread as is the under-representation of older people and people with HF [5–7, 12, 16]. The proportion of patients with HF was significantly higher in Cohort 2 than Cohort 1 of both the observational study and service evaluation. The proportions of patients with HF referred and entering CR in Cohort 2 were higher than those recently reported in the NACR 2014 and 2015 audit data for England (2.8% and 4.4% respectively) [12, 16]. Overall, the proportion of referred patients entering CR was significantly higher in Cohort 2 compared with Cohort 1. Taken together these results support a beneficial effect of the CR service redesign in increasing referral and entry of patients into CR, and increasing recruitment to CR of patients with HF. The mean age of patients referred for CR was similar in cohorts 1 and 2 and similar in patients entering and declining CR in Cohort 1, however, in Cohort 2 those entering CR were approximately 5 years younger than those who declined CR. The results in Cohort 2 are broadly consistent with recent NACR reports indicating that patients entering CR were 8–9 years younger than the overall patient population eligible for CR [12, 16].

Systematic reviews and meta analyses of CR clinical trials indicate that significant reductions in readmissions in patients participating in CR, compared with those not participating in CR, tend to be observed only in the short-term, up to 12 months of follow-up [5–7]. The observed reduction in patients with ≥1 cardiac-related emergency hospital readmissions within 6 months of discharge and trend towards a reduction in 12-month readmissions in Cohort 2 (overall and in the sub-group of patients entering CR) in this study is therefore encouraging, demonstrating an incremental benefit of the new CR model. These findings may suggest that the short term reduction in emergency readmissions associated with CR may reflect, at least in part, an influence of contemporaneous active participation in CR and the associated contact with healthcare professionals, which may encourage and motivate beneficial lifestyle changes in the short term. These benefits may be lost once regular interactions cease and patients are discharged to long term management. However, given the substantial improvement in patient outcomes over the past three decades due to major advances in medical management and availability of exercise-based CR programmes, the demonstration of an incremental benefit of the novel CR service model highlights the importance of CR service review, re-design and innovation and the results that can be achieved within a single centre.

For patients who entered CR, the absolute proportion completing CR, defined as patients completing an end of programme assessment consistent with BACPR recommendations [10], increased by more than 28% following introduction of the new CR service model, mirroring improvements in CR completion reported in the NACR 2015 audit compared with the 2014 audit [12, 16]. The median time from discharge to CR entry in Cohort 2 was 35 days, representing an improvement of 11 days compared with Cohort 1. Given the mixed patient population included in the present study, the time to CR entry for patients in Cohort 2 is broadly consistent with national guidance that CR should begin within 4 weeks of discharge for patients with MI and PCI and within 6 weeks for patients with CABG [4, 16, 21]. For patients completing CR, the median duration of CR in Cohort 2 was 80 days, significantly longer than in Cohort 1, and consistent with National guidelines indicating an ideal programme duration of 12 weeks [4, 10, 16]. Taken together, these data indicate that the CR service redesign led to a substantial increase in patient engagement with CR. This is reflected in the high levels of patient satisfaction with the information provided in preparation for CR and with the overall CR programme upon completion.

### Study limitations

Cohort 1 patient data were collected retrospectively and were reliant on the accuracy of recording in the medical records whereas data for Cohort 2 were collected prospectively and were, therefore, more likely to be complete and accurate. This may have led to an under- or over-estimation of the impact of the novel CR service on patient outcomes due to the potential for under-reporting in Cohort 1. The impact of CR on mortality (overall survival and cardiac-related mortality) could not be evaluated since information on any deaths occurring outside of inpatient care at BHT was not available.

## Conclusions

Implementation of the novel CR service including a comprehensive programme tailored to individual patient needs and with a more patient-centric focus resulted in a short-term reduction in emergency cardiac-related readmissions and sustained improvements in patient referral, CR entry and patient engagement. This study highlights the benefits that can be achieved within a single centre through CR service review and re-design, including the use of innovative technologies to facilitate patient management and increase patient and carer engagement.

## Abbreviations

BACPR: British Association for Cardiovascular Prevention and Rehabilitation
BHT: Buckinghamshire Healthcare NHS Trust
CABG: Coronary artery bypass grafting
CHD: Coronary heart disease
CR: Cardiac rehabilitation
HF: Heart failure
ICD: Implantable cardioverter defibrillator
IQR: Interquartile range
MI: Myocardial infarction
NACR: National Audit of Cardiac Rehabilitation
NSTEMI: Non-ST-segment elevation myocardial infarction
PCI: Percutaneous coronary intervention
SD: Standard deviation
STEMI: ST-segment elevation myocardial infarction

## References

[1] World Health Organisation. WHO Cardiovascular Diseases: Factsheet 317. 2015. http://www.who.int/mediacentre/factsheets/fs317/en/. Accessed 11 Jan 2017.

[2] Nichols M, Townsend N, Scarborough P, Rayner M. European Cardiovascular Disease Statistics 2012. 2012. https://www.bhf.org.uk/publications/statistics/european-cardiovascular-disease-statistics-2012. Accessed 11 Jan 2017.

[3] Townsend N, Bhatnagar P, Wilkins E, Wickramasinghe K, Rayner M. Cardiovascular Disease Statistics 2015. British Heart Foundation; 2015. https://www.bhf.org.uk/publications/statistics/cvd-stats-2015. Accessed 11 Jan 2017.

[4] Heran BS, Chen JM, Ebrahim S, Moxham T, Oldridge N, Rees K, et al. Exercise-based cardiac rehabilitation for coronary heart disease. Cochrane Database Syst Rev. 2011;CD001800. http://www.cochrane.org/CD001800/VASC_exercise-based-rehabilitation-coronary-heart-disease. 10.1002/14651858.CD001800.pub2 PMC4229995 21735386

[5] Sagar VA, Davies EJ, Briscoe S, Coats AJS, Dalal HM, Lough F (2015). Exercise-based rehabilitation for heart failure: systematic review and meta-analysis. Open Heart.

[6] Anderson L, Thompson DR, Oldridge N, Zwisler A-D, Rees K, Martin N (2016). Exercise-based cardiac rehabilitation for coronary heart disease. Cochrane Database Syst Rev.

[7] Anderson LJ, Taylor RS (2014). Cardiac rehabilitation for people with heart disease: an overview of Cochrane systematic reviews. Int J Cardiol.

[8] Smith SC, Benjamin EJ, Bonow RO, Braun LT, Creager MA, Franklin BA (2011). AHA/ACCF secondary prevention and risk reduction therapy for patients with coronary and other atherosclerotic vascular disease: 2011 update. J Am Coll Cardiol.

[9] Perk J, De Backer G, Gohlke H, Graham I, Reiner Z, Verschuren M (2012). European Guidelines on cardiovascular disease prevention in clinical practice (version 2012). Eur Heart J.

[10] British Association for Cardiovascular Prevention and Rehabilitation. BACPR Standards and Core Components for Cardiovascular Disease Prevention and Rehabilitation (2nd Edition). 2012. http://www.bacpr.com/resources/46C_BACPR_Standards_and_Core_Components_2012.pdf. Accessed 11 Jan 2017.

[11] NICE. NICE Commissioning guides: Cardiac rehabilitation services. 2013. http://www.nice.org.uk/guidance/cmg40. Accessed 11 Jan 2017.

[12] British Heart Foundation. The National Audit of Cardiac Rehabilitation Annual Statistical report 2014. 2014. http://www.cardiacrehabilitation.org.uk/docs/2014.pdf. Accessed 11 Jan 2017.

[13] Balady GJ, Ades PA, Bittner VA, Franklin BA, Gordon NF, Thomas RJ (2011). Referral, enrollment, and delivery of cardiac rehabilitation/secondary prevention programs at clinical centers and beyond. Circulation.

[14] Arena R, Williams M, Forman DE, Cahalin LP, Coke L, Myers J (2012). Increasing referral and participation rates to outpatient cardiac rehabilitation: the valuable role of healthcare professionals in the inpatient and home health settings. Circulation.

[15] Dahhan A, Maddox WR, Krothapalli S, Farmer M, Shah A, Ford B (2015). Education of Physicians and Implementation of a Formal Referral System Can Improve Cardiac Rehabilitation Referral and Participation Rates after Percutaneous Coronary Intervention. Heart Lung Circ.

[16] British Heart Foundation. The National Audit of Cardiac Rehabilitation Annual Statistical report 2015. 2015. http://www.cardiacrehabilitation.org.uk/docs/BHF_NACR_Report_2015.pdf. Accessed 11 Jan 2017.

[17] Kaiser M, Varvel M, Doherty P. NHS Improvement - Heart. Making the case for cardiac rehabilitation: modelling potential impact on readmissions. 2013. http://www.natcansat.nhs.uk/dlhandler.ashx?d=pubs&f=Case_for_CR.pdf. Accessed 11 Jan 2017.

[18] Dalal HM, Doherty P, Taylor RS (2015). Cardiac rehabilitation. BMJ.

[19] Clark AM, Catto S, Bowman G, Macintyre PD (2011). Design matters in secondary prevention: individualization and supervised exercise improves the effectiveness of cardiac rehabilitation. Eur J Cardiovasc Prev Rehabil.

[20] Clark RA, Conway A, Poulsen V, Keech W, Tirimacco R, Tideman P (2015). Alternative models of cardiac rehabilitation: a systematic review. Eur J Prev Cardiol.

[21] National Institute for Health and Care Excellence. Myocardial infarction: cardiac rehabilitation and prevention of further MI: Guidance and guidelines. 2013. https://www.nice.org.uk/guidance/cg172#. Accessed 11 Jan 2017. 31891465

[22] Department of Health. Governance arrangements for research ethics committees: A harmonised edition. 2012. https://www.gov.uk/government/publications/health-research-ethics-committees-governance-arrangements. Accessed 11 Jan 2017.

